# Forecasting Strong Subsequent Earthquakes in Greece with the Machine Learning Algorithm NESTORE

**DOI:** 10.3390/e25050797

**Published:** 2023-05-13

**Authors:** Eleni-Apostolia Anyfadi, Stefania Gentili, Piero Brondi, Filippos Vallianatos

**Affiliations:** 1 Section of Geophysics-Geothermics, Department of Geology and Geoenvironment, National and Kapodistrian University of Athens, 15784 Athens, Greece; elenanif1305@gmail.com (E.-A.A.); fvallian@geol.uoa.gr (F.V.); 2 Institute of Physics of Earth’s Interior and Geohazards, UNESCO Chair on Solid Earth Physics and Geohazards Risk Reduction, Hellenic Mediterranean University Research & Innovation Center, 73133 Chania, Greece; 3 National Institute of Oceanography and Applied Geophysics-OGS, 33100 Udine, Italy; pbrondi@ogs.it

**Keywords:** NESTORE, machine learning algorithm, aftershocks, features, Greek seismicity, clusters, forecasting, training procedure

## Abstract

Aftershocks of earthquakes can destroy many urban infrastructures and exacerbate the damage already inflicted upon weak structures. Therefore, it is important to have a method to forecast the probability of occurrence of stronger earthquakes in order to mitigate their effects. In this work, we applied the NESTORE machine learning approach to Greek seismicity from 1995 to 2022 to forecast the probability of a strong aftershock. Depending on the magnitude difference between the mainshock and the strongest aftershock, NESTORE classifies clusters into two types, Type A and Type B. Type A clusters are the most dangerous clusters, characterized by a smaller difference. The algorithm requires region-dependent training as input and evaluates performance on an independent test set. In our tests, we obtained the best results 6 h after the mainshock, as we correctly forecasted 92% of clusters corresponding to 100% of Type A clusters and more than 90% of Type B clusters. These results were also obtained thanks to an accurate analysis of cluster detection in a large part of Greece. The successful overall results show that the algorithm can be applied in this area. The approach is particularly attractive for seismic risk mitigation due to the short time required for forecasting.

## 1. Introduction

It is widely known that large earthquakes are followed by other earthquakes, usually smaller and occurring in close proximity, days to years later, and that it takes some time for seismicity to return to normal levels [1,2,3,4]. However, it may happen that the following earthquake magnitude is comparable with the previous one. Moreover, aftershocks can affect numerous facilities in a city, and repeated earthquakes worsen the damage already inflicted upon vulnerable structures and infrastructure. Greece’s location at the point of contact between the tectonic plates of Eurasia and Africa has resulted in several geodynamic processes and high seismicity, with multiple events of large magnitude recorded both in ancient and modern times. Greece ranks sixth in the world and first in the Mediterranean region for seismic energy emission [5,6]. The significant geotectonic phenomena, such as the continental convergence, where the oceanic crust of the North African plate is subducted under the European plate, are often associated in the literature with the high seismic activity in Greece. This migration was accompanied by significant crustal shortening and an uplift rate of a few millimeters per year along the Hellenic Arc because of the accretion of African plate sediments beneath the underlying Aegean plate. The rollback of the subducting African slab, resulting in high-rate extension in the back-arc region, is also a significant seismic source. Last but not least, seismic activity is caused by the North Aegean Trough (NAT), the most notable tectonic feature of the North Aegean Sea and the Cephalonia Transform Fault Zone (CTFZ) [7].

The Mediterranean and Greek regions are particularly well-known both for intense seismicity and the large-scale earthquakes that have taken place both in recent years and in ancient times. A typical example is the devastating 365 earthquake of Crete, estimated to have had a moment magnitude of 8.5 or greater. The earthquake is believed to have caused the island of Crete to rise by nine meters, while a tsunami that followed the earthquake destroyed the southern and eastern Mediterranean coasts [8]. On 3 April 1881, the deadliest earthquake (M_w_ 6.5) in Greece’s seismic history devastated the SE Chios island. Numerous fatalities occurred, and the vast majority of facilities were totally destroyed [9,10]. The Great Kefalonia earthquake, which devastated the southern Ionian Islands in Greece in 1953 with a magnitude of Ms 7.2 and killed over 500 people, was another significant earthquake of the 20th century [11]. Among the earthquakes used for the analysis in this article, some are major and particularly important, such as the M_w_ 6.4 Aigio (15 June 1995) [12], the M_w_ 6.5 Andravida (8 June 2008) [13], the M_w_ 6.9 Limnos (24 May 2014) [14], the M_w_ 7.0 Samos (30 October 2020) [15], the M_w_ 6.3 Elassona (3 March 2021) [16], and the M_w_ 5.7 Arkalochori (27 September 2021) [17] earthquakes.

In such seismotectonic context, to help mitigate the seismic risk after a strong earthquake, it may be useful to develop and test an algorithm, based on the immediate mild aftershocks, for forecasting the occurrence of stronger subsequent earthquakes. For real-time or near-real-time applications carried out during a cluster occurrence, it is not known whether a first high-magnitude earthquake will be followed by one or more strong events. For this reason, we use the term “o-mainshock” (short for “operative-mainshock”), which refers to the first earthquake in the cluster that exceeds a certain magnitude threshold [18].

Many studies have focused on the value of *Dm*, which corresponds to the magnitude difference between the mainshock and the Strongest Subsequent Large Earthquake (SSLE) [19,20,21,22,23,24]. The magnitude of the SSLE increases as *Dm* decreases, making the cluster more dangerous for the population. Using the assumption of the self-similarity theory of seismicity, which assumes similar behavior for shocks of different magnitudes, studies on this topic are based on *Dm* rather than SSLE magnitude. This approach also has the advantage of using clusters characterized by mainshocks of lower magnitude, which are more frequent than others, and thus, by increasing the training and testing database, improve statistical reliability. Some studies investigating the relationship between *Dm* and mainshock characteristics show that they vary considerably depending on the region [18].

In this paper, we propose a machine learning approach to the problem of *Dm* fore-casting during the occurrence of seismic clusters. NESTORE (NExt STrOng Related Earthquake) is a machine learning-based approach for SSLE forecasting that can be applied to clusters whose magnitude of completeness is at least equal to the mainshock magnitude minus 2 [18,25,26,27]. The clusters are divided into two groups based on the mainshock magnitude *M_m_* and the SSLE magnitude: Type A if *Dm* ≤ 1 and Type B otherwise. The method is based on the analysis of the seismicity after the mainshock by extracting some features used for machine learning. The features describe the characteristics of the seismicity during the cluster in terms of radiated energy, number of events, and space and time distribution. NESTORE trains a one-node decision tree for each feature separately and evaluates thresholds so that clusters whose feature is above the threshold are classified as Type A and the others are classified as Type B. The probability of being a Type A cluster is independently estimated for each feature classifier from the percentage of Type A clusters below and above the threshold in the training set; these probabilities are combined for a final probability estimate using a Bayesian approach [25]. To simulate the increase in knowledge over time after the o-mainshock, the analysis was performed at different time intervals T_i_, ranging from 6 h to 7 days after the mainshock. In this case, we applied the NESTORE algorithm to the Greek seismicity by using the NESTOREv1.0 software available on GitHub [26].

## 2. Geology and Tectonics

**Figure 1:**
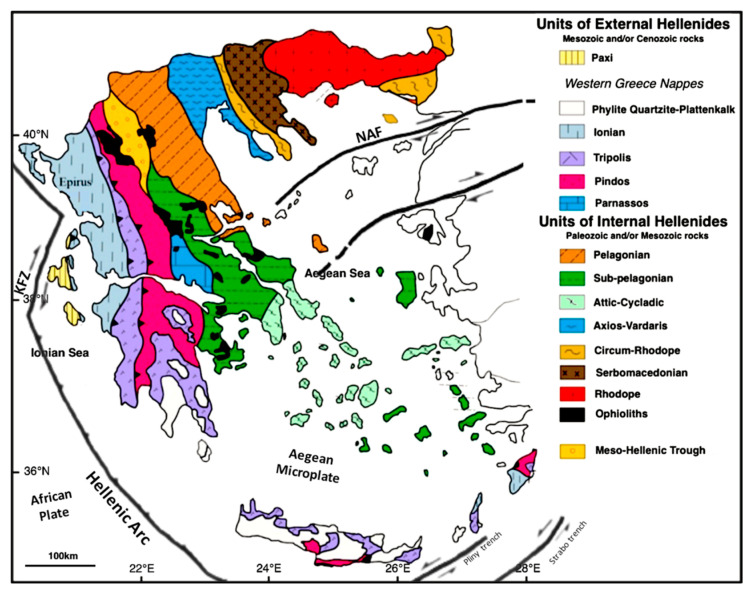
Simplified geotectonic map of Greece modified after [43] which shows the North Anatolian Fault (NAF), the Kefallonian Transform Zone (KFZ), and the Hellenic Arc.

Greece is a typical region of Neo-Europe and is associated with the Alpine orogenic system, which includes the Hellenides. The subduction of the African plate under the Eurasian plate defines the Hellenic Arc system, and the complex process of detachment at the top of the orogenic arc forms the numerous tectonic units of the Hellenides (see Figure 1) [28]. The most recent evolutionary stages of Greece are represented by the Ionian and Paxi geotectonic units, whose rocks are overthrust blocks of the external Hellenides with limestones, schists, and dolomites. The Peloponnese peninsula includes several geotectonic units, such as the Ionian and the Pindos units, which is composed of Mesozoic deep-water carbonates and siliciclastic rocks. The Tripolis unit consists of Paleogene flysch sediments and thick Mesozoic shallow-water carbonates, while the Sub-Pelagonian is made up of clastic formations, limestones, dolomites, and in some cases, ophiolitic formations [29,30,31].

Internal and external Hellenides are found throughout central Greece. Attica is located at the easternmost point of Central Greece and is mostly composed of post-alpine formations and alpine basement rocks, both metamorphic and nonmetamorphic. The high-pressure metamorphic units of the Attic-Cycladic (shales, marbles, schists) and the Sub-Pelagonian unit are the origins of the Alpine rocks. Thessaly is part of the Internal Hellenides, with the Pelagonian Massif and Sub-Pelagonian unit [32]. The Rhodope Massif, the Serbomacedonian Massif, the Axios-Vardaris (Vardar Zone), and the Circum-Rhodope Belt are the tectonostratigraphic units that encompass the Halkidiki Peninsula from east to west. The Vadar zone, an extensive belt with NNW and SSE trends, is considered a suture zone due to its numerous ophiolitic bodies [33]. The Serbmacedonian Massif is mainly composed of gneiss and marble in the north. The Rhodope and Circum-Rhodope belts are composed mainly of marble [34]. Crete is formed by the Gavrovo (Tripolis), the Pindos, the Plattenkalk tectonostratigraphic units, and the Phyllite-Quarzite sequence. On the island, limestones, partially recrystallized, are the lowest rocks visible and near-horizontal faults during crustal compression deposited limestones and other rocks of comparable age above [35].

Apart from the Hellenic Trench, the Kefalonian Transform Zone [36], and the North Anatolian Fault (NAF) [37], there is a large number of active faults on both the mainland and the islands, contributing to the release of seismic energy in Greece. More precisely, the Peloponnese and Central Greece are mainly influenced by alpine thrusts and characterized by post-alpine faults [38]. Furthermore, these regions are mainly dominated by active normal faults [39]. Evia is dominated by normal and strike-slip faults that mainly rotate counterclockwise [40]. Thessaly is characterized by an active tectonic regime as well as sporadic earthquakes [41,42]. Crete is part of the Hellenic Arc and was formed by the subduction of the African plate under the Aegean Sea.

## 3. Data and Region Analyzed

Most statistical or machine learning methods require a large training dataset (hundreds of samples). Even though NESTORE is optimized for small datasets [18], it requires a sufficiently large number of Type A and B clusters (tens of clusters) whose magnitude of completeness is at least two magnitudes lower than that of the corresponding mainshocks. To achieve this, the use of an earthquake catalogue with a long time span, a large area, and a low completeness magnitude is essential to obtain an adequate input database. At the same time, data with low location accuracy and volcanic areas should be avoided because the triggering mechanism of earthquakes is different. Considering all these points, several tests were carried out with different available catalogues, and it was found that the Aristotle University of Thessaloniki earthquake catalogue (AUTH) [44] was the most suitable for the time period 1995–2022. This database was also previously used in the study conducted by Bountzis et al. (2022) to identify seismic clusters in specific regions of Greece [45].

For the analysis, we took into account the regions selected by Bountzis et al. (2022) corresponding to the Corinthian Gulf area, the Ionian Islands, and the North Aegean Sea. Bountzis et al. (2022) selected these regions based on factors such as the homogeneity of focal mechanisms and continuous, comparatively intense seismicity. The Corinthian Gulf is characterized by high rates of extensional deformation, and eight significant faults bounding the rift to the south and dipping to the north are mainly responsible for seismicity [46,47,48,49,50]. In the central Ionian Islands, the Kefalonian Transform Fault Zone, which includes the Lefkada and Kefalonian faults and extends for more than 100 km along the western coast of these islands, is the main seismotectonic domain, and the predominant fault type is right-lateral strike-slip motion. The northern Aegean is characterized by a dextral strike-slip fault running through the North Aegean Trough and its parallel branches, which is the result of the westward propagation of the North Anatolian Fault into the Aegean [51,52,53].

In order to extend the available dataset over a particularly seismically active region, we extended the analysis to the area of Crete for the same time period. An area of predominantly oblique motion is located over well-defined detachment zones in southern Crete, while north-dipping thrust faults are found due to the westward propagating Hellenic fold-and-thrust system [54].

**Figure 2:**
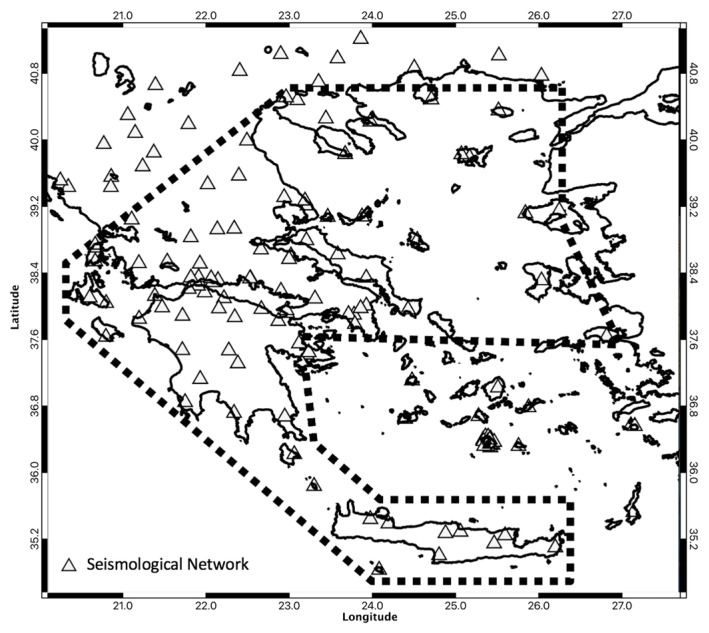
The region we examined in this paper is outlined by the black dashed polygon. The triangles show the locations of the installed Hellenic Unified Seismological Network (HUSN).

In the first tests, we performed the analysis for each region separately to account for the different seismotectonic regimes. However, the results were not statistically relevant due to the small number of clusters in each region. We performed an analysis to check if the clusters belong to the same population and we combined all the above regions into a single area (see Figure 2); we merged the original regions by adding the Peloponnese, Thessaly, Central Greece, and Crete, but omitting (1) the area of the Greek volcanic arc because of the possible different origins of the earthquakes, (2) the area of the subduction zone because of possible viscoelastic effects, and (3) the western Turkish coast and offshore regions because of the poor coverage by the national seismological network.

## 4. NESTORE Algorithm

The multiparameter machine learning approach called NESTORE examines the evolution of seismicity at various time intervals. Its main goal is the estimate of the probability that the analyzed clusters are of Type A. This machine learning approach is designed particularly for the analysis of seismicity problems and limited data, as there are typically tens of available clusters. In order to simulate the evolution of seismicity over time, the analysis was conducted on increasing time intervals *T_i_*, beginning shortly after the mainshock. NESTOREv1.0 uses earthquakes with magnitude M ≥ M_m_ − 2, and to avoid classifications of clusters in which the class has been already defined, it examines Type A clusters for time intervals shorter than the time difference between the mainshock and the first aftershock with magnitude ≥M_m_ − 1. For this reason, both the training and the test sets change depending on the considered time interval, because for longer time intervals, fewer Type A clusters are available.

A set of features (see Supplementary Materials for a detailed description) are extracted from spatio-temporal and energy distribution of seismicity, and for each feature independently, a simple threshold is used to distinguish between the classes. The analysis focuses on features based on the earthquakes following the o-mainshock attempting to capture high and irregular earthquake activity [55,56]. It is important to remark that the framework of the algorithm is independent of the specific features used, which can be adapted based on the study area’s characteristics, including both aspects of seismicity and data availability. Before strong earthquakes, some variations and a change in earthquake flow, which becomes more intense and anomalous in space and time, have been reported and analyzed as a symptom of instability of a nonlinear system equivalent to seismic faults [57]; Vorobieva and Panza (1993) supposed that similar behavior can be detected if a strong subsequent earthquake is expected (Type A clusters) [19]. This is the assumption on which the features adopted by NESTOREv1.0 software package are based. From a physical standpoint, these variances are comprehensible because the lithosphere may respond to tectonic stress more strongly, and since those symptoms appear after a mainshock, they may be precursors to the occurrence of a second major event [58].

The NESTOREv1.0 software package is divided into four main modules. In our analysis, we used the cluster identification, training, and testing modules [26]. The cluster identification module identifies seismic clusters whose mainshock has a magnitude *M_m_* above a given threshold of magnitude *M_th_*. The training module uses decision trees based on different features to find appropriate thresholds with the aim of discriminating clusters of known class (A or B) in a training database. The testing module is used to check the performances of the training; it uses the outputs of the training module to provide an estimate of the probability that a cluster is of Type A in a test database; then it compares the result with the already known actual class of the clusters. The last module, not used in this work, is the near-real-time classification module, which has been proposed recently [26] for new ongoing cluster classifications after validation of the method in an area. Such validation is the topic of this paper.

### 4.1. NESTORE Cluster Identification

Since cluster identification is a non-unique process, there are numerous methods in the literature that provide a range of results. It is easier to solve the task of declustering a catalogue by removing dependent earthquakes than to assign each dependent event to a particular cluster, since this may be controversial for clusters that are close in time and space. The events belonging to each cluster can be significantly affected by the choice of cluster identification procedure. Different methods have been used to identify clusters, depending on the research field. For example, a deep learnable scattering network had been used to cluster seismic events in continuous waveforms [59]. Another method related to distinguishing different structures of lightning phenomena in a multidimensional image developed an analysis pipeline using the t-distributed stochastic neighbor embedding (t-SNE) method and a DBSCAN algorithm for further cluster detection [60]. In space-time analysis of seismicity, many cluster identification algorithms are applied (for further details, see [61]). In particular, in our research, the NESTOREv1.0 cluster identification module implements a simple method of cluster identification, a window-based technique [61], where the cluster is defined as all events occurring within a time and space window around the mainshock, the size of which depends on the magnitude of the mainshock. Window techniques provide a quick and easy way to detect mainshocks and aftershocks, but it is necessary to define a region-dependent law for the maximum time interval *t*(*M_m_*) after the mainshock and the maximum distance *d*(*M_m_*) from the mainshock of the earthquakes inside the cluster [61].

### 4.2. NESTORE Training Procedure

NESTORE assesses a set of features individually before combining the best feature classification after training. In particular, each feature is assessed using a pattern recognition method that employs an independent decision tree [62,63] and the algorithm then determines a threshold *Th* for each feature, *f*, so that if *f* ≥ *Th*, the cluster is designated as A and otherwise as B. The features are calculated at time intervals [*s*_1_, *s*_2_], where *s*_1_ is the time after the mainshock used to guarantee that the completeness magnitude of $M_{c}\leq M_{m}-2$ can be achieved [18] and *s_2_* corresponds to the ending time for the analysis. The features [27] used by NESTORE in this case are evaluated using events having magnitude $M\geq M_{m}-2$ and correspond to nine seismicity parameters related to the number of events, their spatial distribution, magnitude, and energy trend over time in increasing time intervals following the occurrence of the mainshock [18,26]; see the Supplementary Materials for a detailed description.

The major goal of utilizing these features is to spot changes in the flow of earthquakes, such as irregularities in space, time, and magnitude that can be related to different seismic behaviors between Type A and B clusters. In addition, in order to achieve a balance between the need for as many clusters as possible for our study and the necessity for a strong enough statistic on the development of seismicity, we set up the analysis starting at the first 6 h (0.25 days) after the mainshock and at time intervals *T_i_* ending 0.25, 0.50, 0.75, 1, 2, 3, 4, 5, and 7 days after [18,25,26,27,64].

The training set of samples and the expected output class are inputs to the training procedure, which consists of the following sections: feature extraction, decision tree training, good interval identification, inheritance, and validation [18].

**Figure 3:**
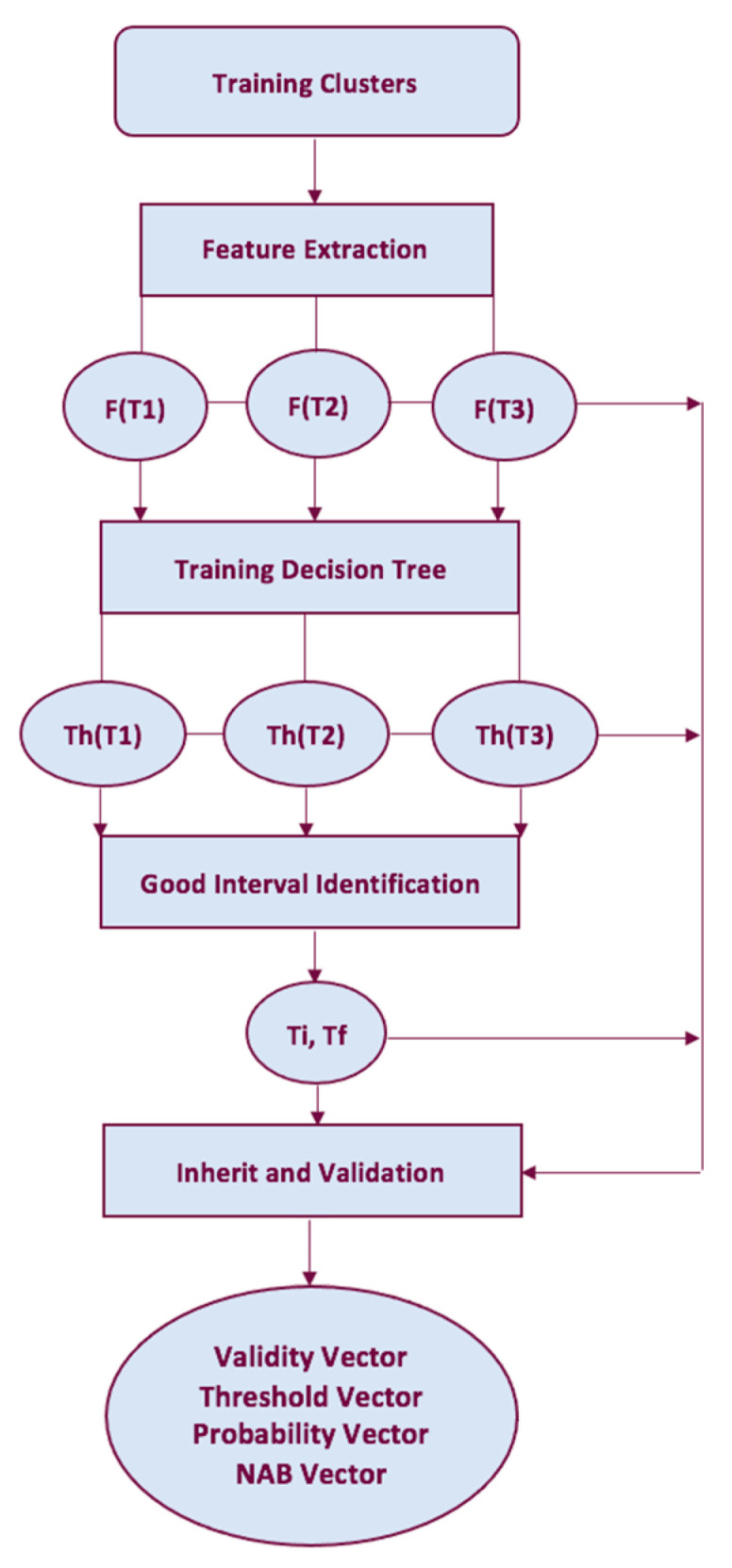
NESTORE training procedure template for a single feature.

NESTORE algorithm is based on a supervised training approach. For each time interval following the mainshock, the algorithm extracts the desired features from the input training clusters (see Figure 3). To prevent a too complex structure of the classifier, which would lead to an overfitting of the data if the data are few, the training is performed using binary decision trees with a depth of 1, which splits classes based simply on a threshold. Using the information on the class of the training clusters, the threshold is chosen such that (most of) the clusters of Type A have features greater than or equal to the threshold, while (most) Type B clusters have values of the features under the threshold. If no tree can be found to solve the issue, the value NaN (Not a Number) is assigned as the threshold and the feature is ignored for that time interval. The performance could be poor even when the decision tree finds a threshold; in order to avoid this problem, the quality of threshold-based classifiers is estimated by performance evaluators.

Specifically, performance evaluators are Accuracy, Recall, Precision, and Informedness. The last evaluator is given between −1 and 1, where 1 is the best and −1 is the worst. The first previous three evaluators are specified between 0 and 1, where 1 is the best and 0 is the worst. As the observation time *T_i_* grows, the value of these performance indicators often increases until it reaches a peak, and then stabilizes or decreases with longer observation time. The algorithm chooses intervals *T_i_* in which three requirements are satisfied. The first states that Recall, Precision, and Accuracy should all be greater than 0.5. The second one specifies that Accuracy should be greater than or equal to the Accuracy we can obtain from a constant response corresponding to the most populous class (Class B). The last one states that Informedness should be greater than zero. Therefore, for each time interval, a set of reliable classifiers corresponding to a subset of the original features is selected.

When the greatest level of Informedness is reached for a feature at a particular *T_i_*, the instances of that feature for each cluster of NESTORE are automatically set to the value they have for *T* = *T_i_.* The time intervals smaller than or equal to *T*_i_ that satisfy the previous three conditions are called good intervals. For longer time intervals, both the feature value and its threshold are set to the ones corresponding to the maximum value of Informedness. This procedure, called inheritance, is intended to use features with high performance in a given time period, even for longer periods when performance is becoming poorer. However, as *T_i_* increases, inherited features and thresholds may experience a fall in performance due to a selection effect on clusters, since for some features, Type A clusters with later SSLEs belong to a separate population. Over time, the percentage of these clusters in the dataset rises, resulting in a decline in feature performance. For this reason, the algorithm rechecks the performances. It determines if the percentage of Type A clusters properly categorized is higher than the percentage of Type B clusters mistakenly classified as Type A, for all inherited thresholds and features. If this does not happen, the interval *T_i_* is removed from the list of intervals associated with the feature [18].

### 4.3. NESTORE Testing Procedure

**Figure 4:**
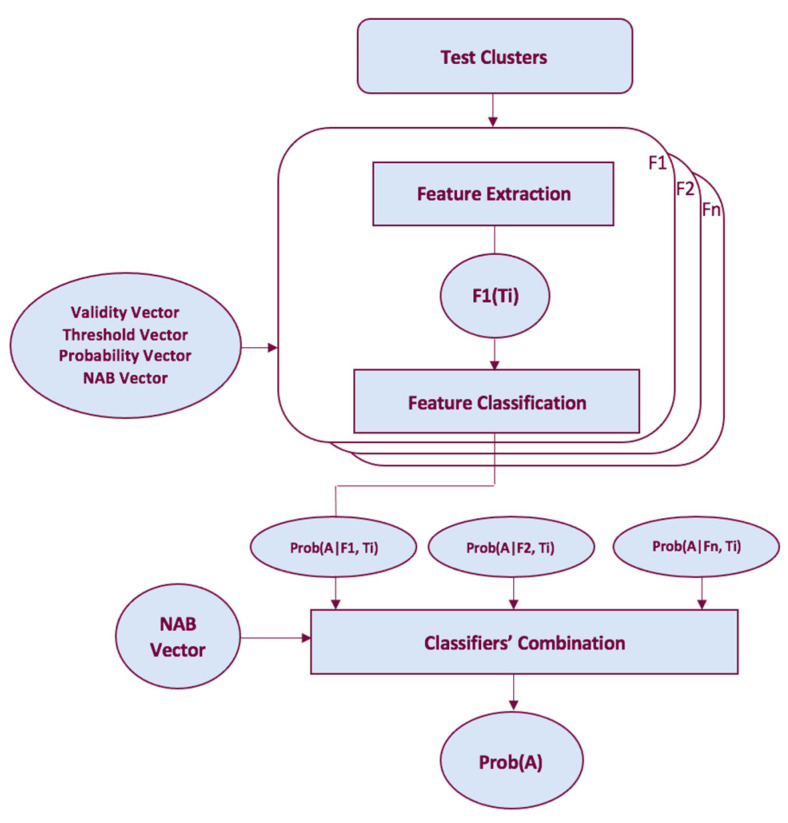
Classification procedure for a time interval *T_i_*.

The NESTORE testing procedure (see Figure 4) uses the information from the training procedure to classify clusters of an independent test set and compares the obtained results with the actual value of the cluster typology. The classification is performed for all the time intervals and for all the classifiers considered reliable in those time intervals by the training procedure. For each time interval, combining the different classification results, a unique classification is produced, which can be binary (“class A” or “class B”) or continuous (“class A probability”). A voting process is the simplest method for classifying combinations, where each classifier receives one vote (A or B). If the number of A votes exceeds the number of B votes by a certain amount, the classification is A; otherwise, it is B. The previous methods presuppose that all classifiers are equally reliable, but in reality, this is frequently not the case. Therefore, for each time interval and feature, NESTORE estimates the probability that a cluster belongs to Type A depending not only on whether it is above or below the corresponding threshold *Th* for single features, but also on how reliable each feature classification is above or below *Th*. By combining many independent classifiers, NESTORE uses a Bayesian technique to determine the total probability [25].

According to Bailer-Jones et al. (2011), utilizing independent information (feature) *D*_1_, …, *D_n_*, the posterior probability that class is *C* is [65]:

(1) $$P\left(C|D_{1}\ldots D_{N}\right)=\alpha \frac{\prod_{n=1}^{N}P(C|D_{n})}{P{\left(C\right)}^{\left(N-1\right)}}$$

where *P*(*C*) is the probability of having a class *C* and *P*(*C*|*D_n_*) is the posterior probability that the class *C* is at *D_n_*. *N* is the number of classifiers and *α* is the normalized factor such that

(2) $$\sum_{k}P\left(C_{k}|D_{1},\ldots D_{N}\right)=1$$

where *C_k_* are the classes of the problem.

In our case, we have two classes, A and B and, assuming for each i-th time interval

(3) $$P\left(A\right)=\frac{N\left(A\right)}{N\left(A\right)+N\left(B\right)}\;\mathrm{and}\;P\left(B\right)=\frac{N\left(B\right)}{N\left(A\right)+N\left(B\right)}$$

where *N*(*A*) and *N*(*B*) are the number of *A* and *B* clusters in the dataset for the *i*-th interval, respectively. Equation (1) can be written as

(4) $$P\left(A|D_{1}\ldots D_{N}\right)=\frac{{\left[N\left(B\right)\right]}^{N-1}\prod_{n=1}^{N}p_{n}}{{\left[N\left(B\right)\right]}^{N-1}\prod_{n=1}^{N}p_{n}+{\left[N\left(A\right)\right]}^{N-1}\prod_{n=1}^{N}(1-p_{n})}$$

where $p_{n}=P(A|Dn)$ is the probability of having the cluster of Class A at a *D_n_* value for the feature *n*; *p_n_* is calculated from the training as the percentage of Type A clusters (divided by 100) that are above or below the output threshold and acts as a weight depending on different features’ reliability. A benefit of this method is that it takes into consideration the number of Type A and B clusters in the dataset, which is crucial for imbalanced classes such as the one we have (e.g., the Type A clusters account for one-fifth of the total clusters in Greece). The testing supplies in output the Receiver Operating Characteristics (ROC) and the Precision–Recall graphs, which show the performances of the training on an independent test set (see the Supplementary Materials for a detailed description).

Binary classifiers distinguish between two classes, one positive (in our case, Class A) and one negative (in our case, Class B). To evaluate the effectiveness of single-features classifiers to determine if a strong aftershock would occur within a cluster, the output Prob(A) for each test set cluster is binarized, so that if Prob(A) ≥ 0.5, the class is A; otherwise, it is B. Resulting classes are compared with the actual one for each cluster and the results are shown by using the ROC graph together with the Precision–Recall graph.

The ROC graph (see, e.g., 6th figure (a,c) in Section 5) shows the normalized percentage of positive instances correctly classified as positives (True Positive Rate or Recall) vs. the percentage of negatives incorrectly classified as positives (False Positive Rate). In the ROC graph, a discrete classifier generates some points whose coordinates graphically represent its performances [27]. The ideal classifier is represented by the point (0, 1) when all instances are correctly classified [27]. In fact, if a point in the space ROC is closer to the point (0, 1), it has a higher rate of positive and/or a lower rate of negative results, so it is preferable to other points. The diagonal line indicates random guessing, and any classifier that occurs in the lower right triangle performs worse than the random one and should be discarded.

The Precision–Recall graph (see, e.g., 6th figure (b,d) in Section 5) shows other useful information: the Precision, which corresponds to the percentage (normalized to 1) of clusters classified as A that are actually A. This information is important for evaluating performance on imbalanced datasets. While both Recall and the False Positive Rate are independent on the relative abundance of the classes, the abundance affects Precision and, therefore, the random guessing horizontal line in the Precision–Recall (PR) graph. As there are fewer A’s over longer time periods, the random guessing line parallel to the x-axis has a decreasing y-intercept as T_i_ increases. A classifier that lies below the random guessing line is characterized by unreliable results; the closer it is to the upper right corner, the more effective it is. The ideal classifier, shown by the upper right corner of the PR graph, correctly classifies all Type A clusters and misclassifies no Type B cluster as A. The best performance for the Precision–Recall graph corresponds to point (1,1) [27].

## 5. Results

**Figure 5:**
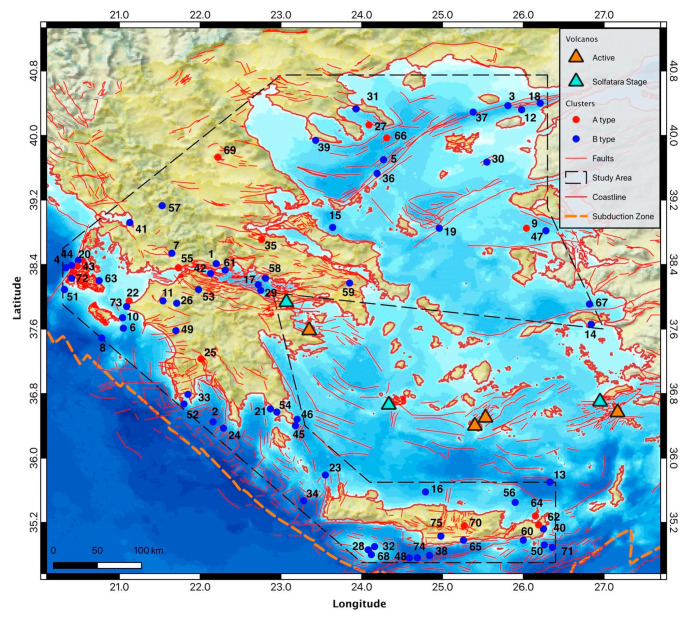
Analyzed region. The mainshocks of the clusters are shown by circles.

In our case study, we applied NESTOREv1.0 to Greek seismicity using the AUTH earthquake catalogue, expressed in magnitude ML, for the period 1995–2022 with a maximum focal depth of 50 km; the analyzed region is shown in Figure 5.

### 5.1. Cluster Identification and Completeness Magnitude Assessment in Greece

In window-based cluster identification applications, the first step is to evaluate how the temporal and spatial extent of the cluster depends on the magnitude of the mainshock. An incorrect assessment may lead to the loss of events belonging to some clusters, thus underestimating their impact on the analyzed area, or, conversely, in including background events or events belonging to other clusters, thus overestimating the impact of the clusters on the area. Since this pre-selection can influence the results of the following analysis, it is an important preliminary step of the cluster analysis. In order to understand which was the most appropriate law for window-based cluster identification in Greece, we compared several laws available in the literature that have been successfully applied to other parts of the world. In these laws, both the duration of the cluster and the radius of a circular area around the mainshock in which aftershocks occur are given as functions of the mainshock magnitude. We set the minimum magnitude of the mainshocks equal to 4, and we tested the equations for duration of Gardner and Knopoff (1974) [66], Lolli and Gasperini (2003) [67], Gentili and Bressan (2008) [68], and Uhrhammer (1986) [69]. For space windows, the equations of Kagan et al. (2002) [70], Uhrhammer (1986) [69], Gardner and Knopoff (2000) [71], and Gentili and Bressan (2008) [68] were tested, the last one with the addition of two kilometers to account for localization inaccuracies.

**Figure 6:**
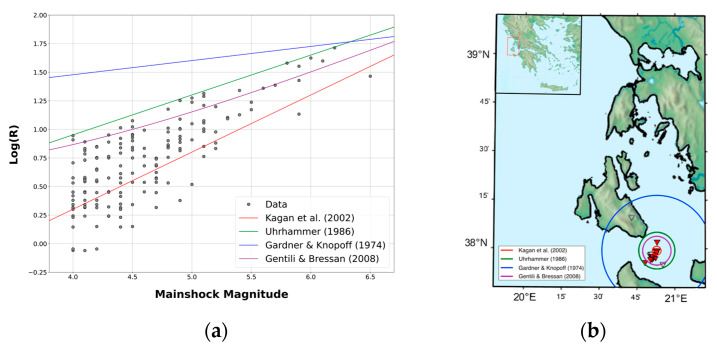
(**a**) Plot of the calculated radius vs. the magnitude of the cluster mainshock. The coloured lines indicate the radius estimation equations. (**b**) Determination of the best space-window law by map visualization [66,67,68,69,70,71].

The choice of the best law for Greece was performed manually. First of all, we estimated manually the distance between the mainshock and the most distant aftershock for a large dataset of clusters and plotted this radius as a function of magnitude, comparing it with the curves representing the equations to be checked. The main idea was to select a curve that corresponds to the smallest radius that encompass most of the clusters, in order not to lose events belonging to the cluster but, on the other hand, to avoid the inclusion of independent events. Figure 6a shows such a plot on 177 clusters in the area.

**Figure 7:**
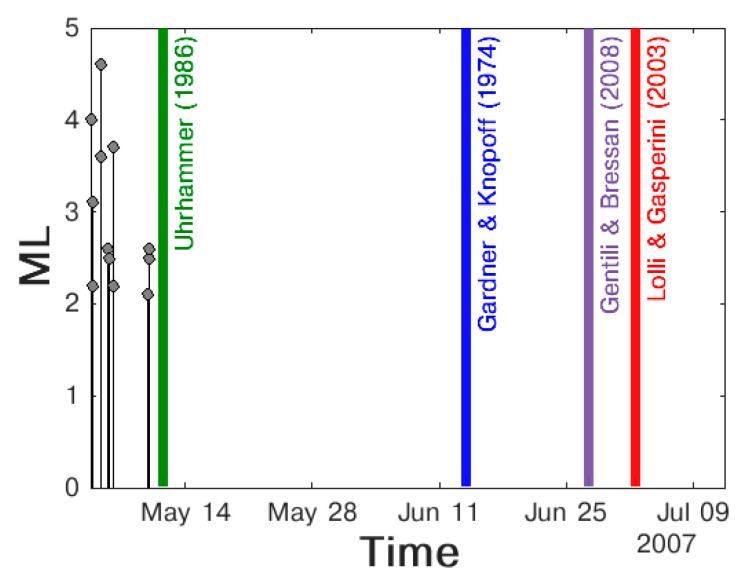
Determination of the best time-window law [67,68,69,71].

In order to check the results on a larger dataset, we also manually inspected the maps of all the clusters obtained by imposing the larger radius equation (Gardner and Knopoff, 1974) [66] and comparing earthquakes’ positions with the circles representing the checked equations (see Figure 6b). In both cases, the best choice was the equation proposed by Uhrhammer (1986) [69]. This equation provides a much smaller radius than the one proposed by Gardner and Knopoff (1974) [66], but also helps to avoid the inclusion of independent earthquakes in a cluster. In addition, it provides a larger radius compared to the equations proposed by Kagan et al. (2002) [70] and Gentili and Bressan (2008) [68], allowing more aftershocks to be included in the defined cluster. For time window, we plotted the magnitude vs. time for the obtained clusters (see Figure 7) and we compared it to the duration obtained by different equations. Again, the best choice was the equation proposed by Uhrhammer (1986) [69], which supplies a shorter *t*(*M_m_*) compared to the other methods (see Figure 7) and has the advantage of including highly dependent events in the cluster.

Equations (5) and (6) show the selected radius (in km) and duration (in days):

(5) $$d=e^{-1.024+0.804Mm}$$

(6) $$t=e^{-2.87+1.235Mm}$$

This procedure failed only in two earthquakes in the northern Gulf of Evia. The first occurred on 17 November 2014 and the second on 9 June 2015, with magnitudes ML of 5.3 and 5.1, respectively. As indicated by Ganas et al. (2016) [72], the above earthquakes belong to the same cluster, which is a Type A cluster according to the NESTORE classification. However, the applied method of cluster identification leads to an obvious classification failure as it splits the cluster into two parts. Since the NESTOREv1.0 module is independent of the others in the NESTOREv1.0 package, it can be substituted with a different cluster identification procedure. A more reliable cluster identification method will be used in the future for the analysis of the region. In this application, we removed the cluster from the analysis.

**Figure 8:**
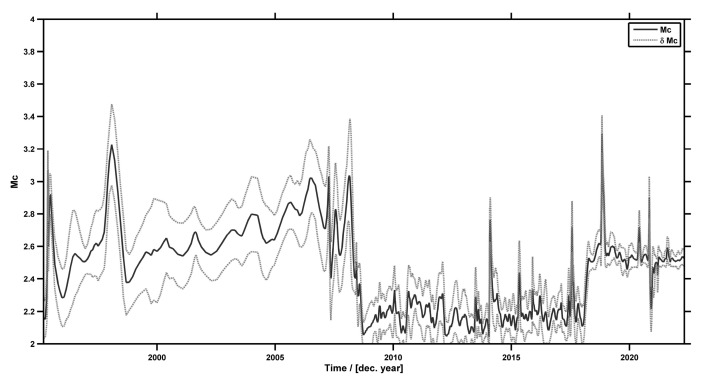
Plot of Mc vs. time for Greece using AUTH earthquake catalogue.

As previously stated, NESTOREv1.0 needs clusters with a completeness magnitude of ≤*M_m_* − 2, where *M_m_* is the o-mainshock magnitude. When at least 80 earthquakes are available in a cluster, NESTOREv1.0 automatically evaluates the completeness magnitude for the cluster using the maximum curvature method (+0.2 to account for possible underestimates of the method); otherwise, it allows a default value. We considered a completeness magnitude value of 3.0 for clusters that occurred before 2009 and a magnitude value of 2.5 for those starting in 2009. This assumption is based on a general analysis of the completeness magnitude as a function of time for the analyzed area that we carried out using Zmap software [73] (see Figure 8).

Besides the selection based on the completeness magnitude, another selection of Type A clusters was performed based on the time of the strongest aftershock: since the first analysis is performed 6 h after the mainshock, NESTOREv1.0 analyzed only the Type A clusters which did not have an aftershock with magnitude ≥*M_m_* − 1 in the first 6 h.

At the end of the selection procedure, we detected 75 clusters satisfying NESTORE requirements, of which 12 are Type A and 63 are Type B. In Figure 5, we superimposed on the map of the studied area the locations of the o-mainshock of the clusters; we used red color for Type A clusters and blue for Type B clusters. The clusters are located both offshore and along the mainland.

Analyzing the characteristics of the clusters, we did not find any correlation between the type of cluster (A or B) and some parameters of the mainshock, such as the focal mechanism [37], location, depth, or magnitude.

### 5.2. NESTOREv1.0 Application to the Current Dataset

**Figure 9:**
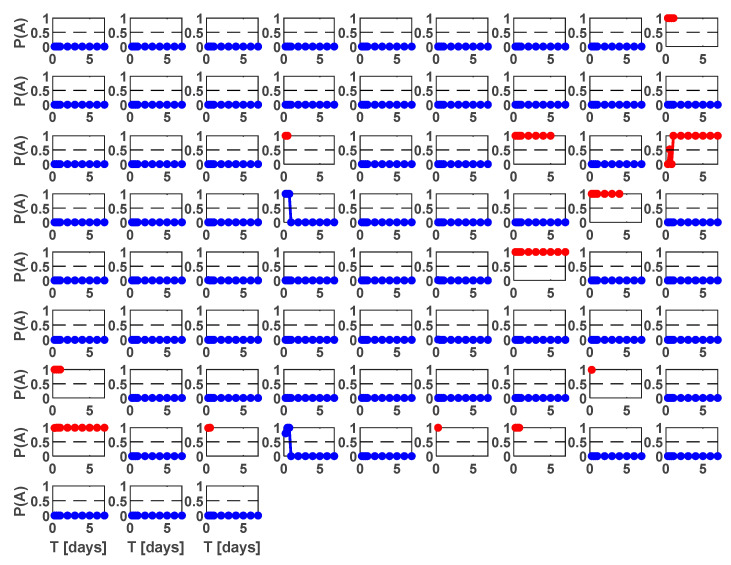
Estimated probability of being a Type A cluster vs. time for all the clusters in the dataset (autotest). Red points correspond to A type clusters, while blue points correspond to B type ones.

As described in Section 2 and Section 3, Greece is extremely heterogenous from a seismotectonic point of view. For this reason, it is important to check that, given one type of cluster (Type A or Type B), all the clusters of that type have similar characteristics (i.e., they belong to the same population) according to the NESTORE model. If there is more than one population depending on the sub-region, the training of each sub-region must be performed separately. In order to check this, we trained NESTOREv1.0 with the whole dataset of 1995–2022 both as a training set and a test set (autotest). Figure 9 illustrates the probability P(A) of being a Type A cluster for different time intervals. The analysis was performed on increasing time intervals ending every 6 h in the first day and every day in the first week after the mainshock.

Each cell corresponds to a different cluster classification for different time intervals. Red circles correspond to Type A clusters and blue ones to Type B clusters.

The figure shows that for most time periods, the probability of being A is close to 1 and close to 0 for B. This is not an assessment of the performance of the method, since overfitting is an obvious risk when the training and test sets are coincident, but a preliminary check of the coherence of the dataset, showing that the two classes can be distinguished and that there are no obvious outliers. In detail, the good result in Figure 9 shows that the clusters of the same type in different parts of Greece belong to the same population from NESTOREv1.0’s point of view, and the analysis can be performed on the whole area together.

To fully exploit the potential of the machine learning approach for Type A cluster forecasting, we created a test set separate from the training set that contains instances with known classes. In the testing procedure, the class of each cluster in the test set is evaluated using the information obtained from training. The forecasted cluster class is compared to the already known actual class to obtain an estimate of the training performance. The choice of the number of clusters to be selected for the training set and the test set could, in principle, affects the results. Especially when few data are available, it is important from one side to have enough data in the training set to have a good estimate of the parameters, but on the other hand to have enough data in the test set, such that the obtained performances are reliable. A rule of thumb often used in machine learning suggests that three-quarters of the total data should be used for training and the remaining one-quarter for testing [74]; however, the number of Type A clusters is only 12 in the dataset 6 h after the mainshock. This means there are only three clusters to check the results for a time interval of 6 h, and fewer for longer time periods, due to the decrease in the number of Type A clusters. Xu and Goodacre proposed a range between 50% and 70% [75], which is more suitable for our application. We used the years from 1995 to 2015 for the training and the following 7 years for testing (see Table 1). Table 1 shows in detail the number of clusters, particularly Type A clusters, in the training set and the test set.

**Figure 10:**
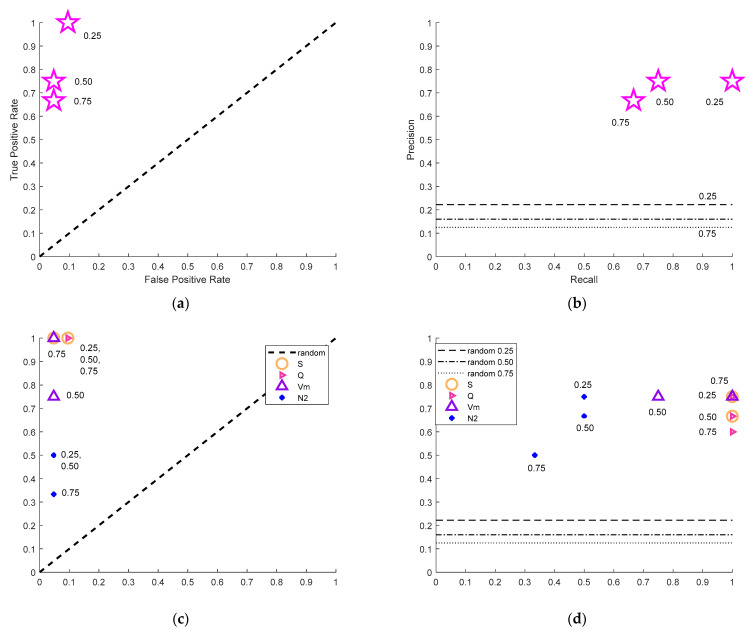
NESTOREv1.0 performances for different *T_i_* values which are listed close to the corresponding star. (**a**) ROC graph for NESTOREv1.0 Bayesian classification; (**b**) Precision–Recall (PR) graph for NESTOREv1.0 Bayesian classification; (**c**) ROC graph for selected features; (**d**) PR graph for selected features.

Figure 10 shows the performances of the method. The NESTOREv1.0 Bayesian classification performance for each time period *T_i_* is shown by magenta stars, and some examples of single-feature classifier performances are shown with different symbols.

**Figure 11:**
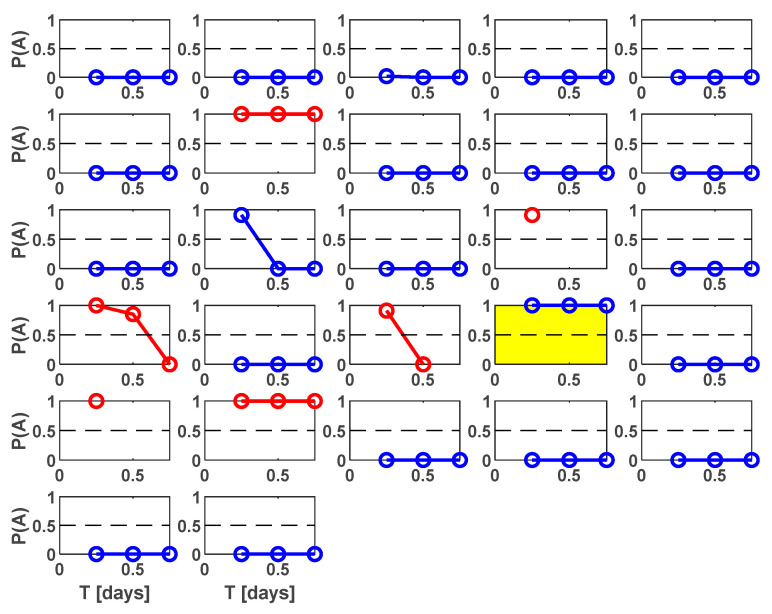
Estimated probability of being a Type A cluster vs. time for all the clusters in the time period 2016–2022 (training period 1995–2015).

Figure 11 shows the probability vs. time of being a Type A cluster obtained for different time periods Ti for the clusters of the test set.

Both the ROC and PR plots in Figure 10a,b show that NESTOREv1.0 Bayesian classification consistently lies within ranges corresponding to reliable classifiers for all time periods, above the random rate line in the PR plots and in the top left triangle for the ROC plots. Longer time periods correspond to a small number of Type A clusters, due to the elimination from the dataset of clusters that already had strong aftershocks. This affects the capability of both the training set and the test set to accurately describe the characteristics of the clusters, and thus, the reliability of results. Therefore, the analysis was stopped at *T_i_* = 0.75 days (18 h) so that we have at least three Type A clusters both in the training set and in the test set. The best performance for both ROC and PR graphs is at 6 h. We hypothesize that this is because, by including more data in the training set and using a more balanced dataset, we were able to better model the complexity of Greek seismicity, allowing the decision trees to converge to a more stable result. The good performances’ short time intervals after the mainshock are noteworthy for the seismic risk mitigation assessment.

Figure 10c,d illustrates the characteristics of some features, selected because of their different performances, to illustrate the whole method procedure. They are calculated for different time intervals and are the normalized cumulative source area (*S*), the normalized radiated energy (*Q*), the cumulative variation of magnitude between each occurrence (*V_m_*), and the number of events (*N*_2_). From one feature to another, different time intervals *T_i_* were needed to achieve the best performance. For shorter intervals following the mainshock, not all of the features were considered reliable or could be computed. Six hours after the mainshock, the feature *Q*, *S*, *Z*, and *N*_2_ are considered reliable, but only two of them supply high-performance results. Comparing feature performances in Figure 10c,d, it can be seen that the features *S* and *Q* produced the best performances, the feature *N*_2_ produced the poorest, and the feature *V_m_* had intermediate results. These differences are mainly related to the smaller recall (True Positive Rate) of these features, very low especially for *N*_2_ (blue dots). At 6 h after the mainshock, the NESTOREv1.0 Bayesian performances coincides with *Q* and *S* feature ones, with a True Positive Rate of 1 (all Type A clusters correctly classified), a False Positive Rate of 0.095 (90.5% of Type B clusters correctly classified), and a Precision of 0.75 (75% of the clusters classified as Type A were actually A). This result corresponds in Figure 11 to two Type B clusters wrongly classified: one in the third row and second column, correctly classified for longer time periods, and the cluster in the fourth row and fourth column, automatically outlined in yellow by NESTOREv1.0 as an outlier, because it supplies a wrong classification in all the analyzed time periods.

In order to evaluate the best value of the threshold for future application of the method to Greek seismicity (by using the near-real-time classification module), we used the ones obtained during the autotest. Since, using all the data, we have no independent test set to evaluate the performances, we stopped our analysis at *T_i_* = 18 h, as in the test shown in Figure 10. Table 2 shows the values of the thresholds for the training set of the autotest at these time intervals. It is noticeable that the larger training set eliminates the poorly performing feature *N*_2_ from the classification at 6 and 12 h.

Table 3 shows the values of *p_u_* and *p_o_* that are used to evaluate *p_n_* of Equation (4): if the cluster is under the threshold, *p_u_* is used; otherwise, *p_o_* is used.

**Figure 12:**
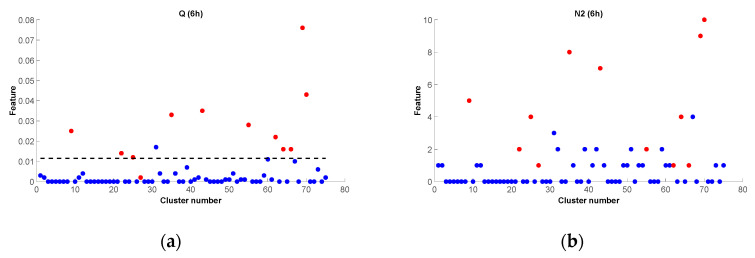
Values of the features Q (**a**) and N2 (**b**) at the 6 h (6 h) time interval for the whole 1995–2022 dataset. Red circles: Type A clusters; blue circles: Type B clusters; black dashed line: the threshold obtained by NESTOREv1.0.

Figure 12 shows a comparison between the features *Q* and *N*_2_ for the 6 h time interval. The clusters are ordered in time, so the circles with cluster numbers from 1 to 46 are the ones from the 1995–2015 training set. It is noticeable how the A clusters can be clearly discriminated from the B ones using the *Q* feature, while *N*_2_ shows mixed classes. In particular, several Type B clusters show a number of events *N*_2_ equal to 2 at 6 h, while there are Type A clusters with a smaller or equal number of events. The bad performances of the *N*_2_ feature in Figure 12 can be explained with the attempt of the algorithm to discriminate the two classes setting high values of the threshold (in this case, 3.50). This choice supplied poor results for the testing of Figure 10c,d because half of the Type A clusters in the test set are under the threshold.

## 6. Discussion

In analyzing Greek seismicity from the perspective of Type A and B cluster analysis, several interesting results emerged that distinguish the seismicity of the area from that of other regions of the world. The first interesting result is that the percentage of Type A clusters in Greece is very low, even considering the smallest time interval analyzed after the mainshock (6 h). In fact, for a time interval of 6 h, the number of Type B clusters is about five times higher than the number of Type A clusters. This number is very high when compared with Italy, northeastern Italy and western Slovenia, and California, where NESTORE has already been applied [18,25,27], where the number of Type B clusters is between 1.5 and 2 times the number of Type A clusters. Moreover, there are no correlations between the cluster type and the focal mechanism, focal depth, magnitude, and location of the mainshock, as observed in some cases in other regions [27].

Previous studies in California [18], Italy [27], and northeastern Italy and western Slovenia [25], corresponding to very different seismotectonic regions, have shown good performance of classifiers based on the number of events (feature *N*_2_) shortly after the mainshock [18]. Classifiers based on the features *Q* and *S* perform well in Italy and western Slovenia, while in California, they provide reliable results only some days after the mainshock. Conversely, feature *N*_2_ gives the worst results in Greece, while features *Q* and *S* give the best results. The difference in performance between features *Q* and *N*_2_ in Greece can be clearly seen in Figure 12. The main difference is related to a large number of Type A clusters with a similar number of aftershocks as B clusters. A further comparison of the *N*_2_ feature at 6 and 18 h, shows that the performance of the *N*_2_ feature improves at longer time periods, since the Type A clusters, characterized by an early strong aftershock and therefore removed from the dataset, are precisely those with a low number of events. These strong early aftershock clusters are not very productive in terms of the number of aftershocks, but they are still productive in terms of the energy of the aftershocks and can therefore be discriminated from B clusters using the features *Q* and *S*, which are related to the magnitude of the aftershocks. This fact and the low percentage of A clusters make us hypothesize that there may be fewer high-energy earthquakes in Greece for the same total energy radiation. This hypothesis is beyond the scope of this paper and should be verified in future work.

Another interesting feature is *QLcum*, which corresponds to the deviation of *Q* from the long-term trend. This feature gives good short-term results after the mainshock for California as well as for northern Italy and western Slovenia, while it requires longer time intervals for Italian seismicity. The numerical values of this feature can only be compared with the application for California, since the interval start time and completeness requirements have changed from previous work. However, it is interesting to note that the thresholds of the other features defined in both Greece and California are similar, with variations within 25%, while the threshold of the *QLcum* feature in Greece is about 12 times higher than that in California. This could be related to the strong temporal variations in the radiated energy in both the Greek Type A and Type B clusters.

For this NESTOREv1.0 application, the performance is good at 6 h and deteriorates over time for longer periods. This trend is explained by the fact that shorter time intervals have a higher percentage of Type A clusters than longer time intervals, where performance is affected by the imbalance of A and B classes, resulting in a reduced ability of the classification system to distinguish between classes. In addition, the effects of background seismicity and activation of nearby fault segments, especially in case of large earthquakes, may reduce the reliability of the features. Importantly, the improved performance shortly after the mainshock is a notable advantage for the application of the algorithm for risk mitigation purposes.

## 7. Conclusions

The NESTORE machine learning algorithm, implemented in the NESTOREv1.0 software package [26], was applied to Greek seismicity to forecast the occurrence of a strong earthquake after an intense mainshock. We used the AUTH earthquake catalogue between 1995 and 2022 over a large area of Greece, consisting of the Gulf of Corinth, the Ionian Islands, the northern Aegean Sea, Thessaly and central Greece, Crete, and the Peloponnese, in order to obtain a long time period and a large area for analysis, and thus to analyze a sufficiently large number of clusters. Using a window-based approach, in which a cluster is defined as all events occurring within a temporal and spatial window around the main earthquake, we tested several laws for cluster detection and found that Uhrhammer’s (1986) [69] law was the most appropriate for identifying clusters in Greece.

NESTORE classifies clusters into two classes, Type A or Type B, depending on the magnitude of the strongest aftershock. The algorithm analyzes seismicity features at increasing time intervals from the mainshock using a training procedure based on single-node decision trees (one threshold for each feature) and found statistically validated thresholds for the features to discriminate the two typologies. After training, a testing procedure estimates the probability for each feature to be a Type A cluster on an independent test set. The estimated probabilities from the different features are combined using a Bayesian approach to obtain the NESTORE response, which takes into account the different degrees of reliability of each feature.

The NESTOREv1.0 cluster identification module is independent of the other two. It allows the user to choose the equations for the radius and time interval of the cluster. This approach allows fitting to different regions for which different equations should be used. However, if a more accurate cluster evaluation procedure is required for a particular region, this module can be modified without affecting the following two modules.

The training and testing modules can be applied to clusters whose magnitude of completeness is at least equal to the magnitude of the mainshock minus 2. The modules require a dataset of tens of clusters for reliable training and testing. Thus, the success of the application of the NESTORE algorithm is influenced by the earthquake catalogue: if the completeness magnitude is too large, and thus, the number of clusters that can be analyzed is too small, the algorithm cannot be successfully applied. In addition, the performance of the features can be affected by the quality of the catalogue used and the magnitudes and the epicenters of the earthquakes. For this reason, a well-covered seismological network is important. To avoid too few clusters or problems related to changes in seismicity over time, the use of data over a period longer than 10–20 years is strongly recommended to cover the variability of seismicity features. Considering these catalogue property requirements, the algorithm has been shown to be robust enough to be applied in different seismotectonic environments. Crucial to this are the training procedure, which allows the algorithm to automatically adapt to the study area, and the clustering approach, which allows different region-specific equations as input.

In our work, we carried out the analysis by NESTOREv1.0 on 75 clusters reported in the AUTH earthquake catalogue from 1995 to 2022, using a training set from 1995 to 2015 and a test set in the following 7 years. In particular, by using ROC and Precision–Recall plots, we show that NESTOREv1.0 provided good performances in terms of Type A clusters forecasting. The best performance was obtained for a time interval of 0.25 days (6 h) after the o-mainshock. Notably, 100% of Type A clusters were forecasted correctly, the percentage of Type B clusters misclassified as Type A clusters was less than 10%, and the percentage of correct classifications was 92%. This makes the method particularly attractive for application in the field of seismic risk mitigation, as it allows estimating the probability of a future hazardous earthquake occurring after an initial strong event.

Our understanding of the SSLE preparation process can benefit from a detailed examination of the features and time periods in which they are relevant to the cluster classification. In particular, the features *S* and *Q*, both depending on the earthquake’s magnitude, perform well shortly after the mainshock, while *N*_2_, depending on the number of earthquakes, performs poorly. Interestingly, in a previous application of the code to California, Italy, and northeastern Italy and western Slovenia, in [18,25,27], *N*_2_ performed best, while *Q* and *S* feature performances depended on the analyzed region.

It is important to remark that NESTORE performs well independently on different regional characteristics because is based on a region-dependent training and because it is based on different features of seismicity. In our opinion, such an approach based on multiple features is pivotal to develop a robust algorithm able to work in different regions.

## Figures and Tables

**Table 1:** Training and testing dataset information.

Training Period	Testing Period	No. of Clusters (Training Set)	No. of A Clusters (Training Set)	No. of Clusters (Test Set)	No. of A Clusters (Test Set)
1995–2015	2016–2022	46	6	29	6

**Table 2:** Values of the thresholds of the features obtained by the training procedure on the whole dataset. Inh. = inherited threshold value.

Features	Thresholds		
	Th (6 h)	Th (12 h)	Th (18 h)
S	0.053	0.053	0.084
Z	0.026	0.026	0.026
SLCum		0.056	Inh.
QLCum		2.318	2.318
SLCum2			0.090
QLCum2			2.79
Q	0.012	0.012	0.013
Vm		0.035	0.450
N_2_			2.5

**Table 3:** Values of the probability of being Type A under and over the threshold for the whole dataset.

Features	Thresholds					
	*p_u_* (6 h)	*p_o_* (6 h)	*p_u_* (12 h)	*p_o_* (12 h)	*p_u_* (18 h)	*p_o_* (18 h)
S	0.02	0.85	0.00	0.83	0.02	0.88
Z	0.06	0.64	0.03	0.62	0.02	0.58
SLCum			0.02	0.82	0.02	0.78
QLCum			0.02	0.69	0.02	0.64
SLCum2					0.02	1.00
QLCum2					0.00	0.67
Q	0.02	0.92	0.02	0.90	0.02	0.88
Vm			0.02	0.75	0.02	0.78
N_2_					0.02	0.63

## Data Availability

The NESTOREv1.0 toolbox is available for free download from GitHub at the address https://github.com/StefaniaGentili/NESTORE and the reproducibility package is available on Zenodo https://zenodo.org/account/settings/github/repository/StefaniaGentili/NESTORE. The catalogue used in this paper is the AUTH earthquake catalogue, available online: http://geophysics.geo.auth.gr/the_seisnet/WEBSITE_2005/station_index_en.html (last accessed on 28 July 2022).

## Supplementary Materials

The following supporting information can be downloaded at: https://www.mdpi.com/article/10.3390/e25050797/s1. Figure S1. Confusion Matrix [76,77].
