# Supplementary material for: Forecasting Strong Subsequent Earthquakes in Greece with the Machine Learning Algorithm NESTORE
Source: Entropy (Basel). 2023 May 13;25(5):797. doi: 10.3390/e25050797 (PMC10217232; doi:10.3390/e25050797)
Supplement: Supplementary file 1 [file entropy-25-00797-s001.zip › entropy-2358449-supplementary.pdf]

Supplementary Material

Forecasting Strong Subsequent Earthquakes in Greece with the Machine Learning Algorithm NESTORE

Eleni-Apostolia Anyfadi [1,2], Stefania Gentili [3,*], Piero Brondi [3] and Filippos Vallianatos [1,2]

1 Section of Geophysics-Geothermics, Department of Geology and Geoenvironment, National and Kapodistrian University of Athens, 15784 Athens, Greece; elenanif1305@gmail.com (E.-A.A.); fvallian@geol.uoa.gr (F.V.)
2 Institute of Physics of Earth's Interior and Geohazards, UNESCO Chair on Solid Earth Physics and Geohazards Risk Reduction, Hellenic Mediterranean University Research & Innovation Center, 73133 Chania, Greece
3 National Institute of Oceanography and Applied Geophysics-OGS, 33100 Udine, Italy; pbrondi@ogs.it
* Correspondence: sgentili@ogs.it

1. Selected Features

Herein, we present the features [1, 2] used by NESTORE in this study and the equations that describe them.

- The feature *S* is the normalized event source area, and it is given by

$$S(i) = \sum_i 10^{(m_i - M_m)} \qquad \text{(S1)}$$

where m_i denotes the event's magnitude at that particular time interval. This is the area that the aftershocks occupy when compared to the region that was occupied by the mainshock in this function.

- The *Z* feature corresponds to the linear concentration of aftershocks where the ratio of the average diameter of the aftershock source is divided by the average distance between aftershocks.

$$Z(i) = \frac{mean(10^{0.69 m_i - 3.22})}{mean(r_{ij})} \qquad \text{(S2)}$$

where r_{ij} is the distance between the ith and j^{th} aftershock in general.

- The sum energy of the aftershocks, normalized to the energy of the mainshock, is referred to as the *Q* feature [3].

$$Q(i) = \frac{\sum_i E_i}{E_m} \qquad \text{(S3)}$$

where E_i denotes the energy of the ith aftershock and E_m denotes the energy of the mainshock. The equation of Gutenberg and Richter (1956) yields the energy *E* (in Joules) of an event with magnitude *M* as follows [4]:

$$Log_{10}(E) = \frac{3}{2}M + 4.8 \qquad \text{(S4)}$$

- The cumulative divergence of S from the long-term trend is the $SLCum$ feature. Given the intervals $[s_1, s_1 + dt], [s_1, s_1 + 2dt], \dots [s_1, s_1 + ndt]$ where $s_1 + ndt \leq s_2$ and $s_1 + (n+1)dt > s_2$, $SLCum$ is defined as follows:

$$SLCum(i) = \sum_i abs\left[S(t_i) - S(t_{i-1})\frac{i \cdot dt}{(i-1) \cdot dt}\right] \quad \text{(S5)}$$

  where $S(t_i)$ is S determined at time $t_i = s_1 + i \cdot dt$ and $S(t_i)$ using the time interval $[s_1, t_i]$. This feature responds to sudden changes in S.

- The cumulative divergence of Q from the long-term trend is the $QLCum$ feature. Like $SLCum$, it is defined on increasing windows after s_1. $QLCum$ is defined as follows:

$$QLCum(i) = \sum_i abs\left[Q(t_i) - Q(t_{i-1})\frac{i \cdot dt}{(i-1) \cdot dt}\right] \quad \text{(S6)}$$

  where $t_i = s_1 + i \cdot dt$ and $Q(t_i)$ is Q calculated on the time interval $[s_1, t_i]$.

- The cumulative deviation of S from a sliding window from the long-term trend is represented by the feature $SLCum2$. The interval $[s_1, s_2]$ is divided into smaller intervals $[s_1, s_1 + dt], [s_1, s_1 + 2dt], \dots [s_1, s_1 + ndt]$ and $[s_1, s_1 + dt], [s_1, +dt, s_1 + dt + d\tau], \dots [s_1, +(n-1)dt, s_1 + (n-1)dt + d\tau]$ where $s_1 + ndt \leq s_2$ and $s_1 + (n+1)dt > s_2$. $SLCum2$ is given by:

$$SLCum2(i) = \sum_i abs\left[S([s_1 + (i-1) \cdot dt, s_1 + i \cdot dt]) - S([s_1 + (i-1) \cdot dt, s_1 + (i-1) \cdot dt + d\tau])\frac{dt}{d\tau}\right] \quad \text{(S7)}$$

  where $S[a, b]$ is S estimated throughout the $[a, b]$ time range in general. This feature responds to sudden changes in S as the feature $SLCum$, but differently from $SLCum$, the window does not start at a fixed time close to the mainshock origin time.

- The cumulative deviation of Q from a sliding window from the long-term trend is represented by the feature $QLCum2$ and it is calculated in a manner similar to $SLcum2$ for the Q function. The interval $[s_1, s_2]$ is separated into a set of smaller intervals $[s_1, s_1 + dt], [s_1, s_1 + 2dt], \dots [s_1, s_1 + ndt]$ and another set $[s_1, s_1 + dt], [s_1, +dt, s_1 + dt + d\tau], \dots [s_1, +(n-1)dt, s_1 + (n-1)dt + d\tau]$ where $s_1 + ndt \leq s_2$ and $s_1 + (n+1)dt > s_2$. $QLCum2$ is given by:

$$QLCum2(i) = \sum_i abs\left[Q([s_1 + (i-1) \cdot dt, s_1 + i \cdot dt]) - Q([s_1 + (i-1) \cdot dt, s_1 + (i-1) \cdot dt + d\tau])\frac{dt}{d\tau}\right] \quad \text{(S8)}$$

  where $Q[a, b]$ is Q estimated throughout the $[a, b]$ time range in general. Similar to $SLCum2$, the feature reacts to sudden changes in Q not beginning at a set time near the mainshock starting moment.

- The V_m feature, which is determined by the cumulative variation of magnitude between each occurrence, is

$$V_m(i) = \sum_i |m_i - m_{i-1}| \quad \text{(S9)}$$

  where m_i is the magnitude of the ith event in the selected time interval.

- Feature N_2 is the number of events with $M \geq Mm - 2$.

2. Performance Estimation

Binary classifiers distinguish between two classes, one positive (in our case class A) and one negative (in our case class B). In pattern recognition applications, the confusion matrix, a two-by-two matrix formed by the number of classification results, is used to obtain information about the performance of a binary classifier (i.e., TP, FN, TN, and FP are the number of True Positives, False Negatives, True Negatives and False Positives, respectively). The numbers of the main diagonal reflect the false choices in the different classes, while the numbers along the main diagonal represent the correct choices. Recall, Precision, and Accuracy evaluations are a standard method for assessing a classifier's performance [5].

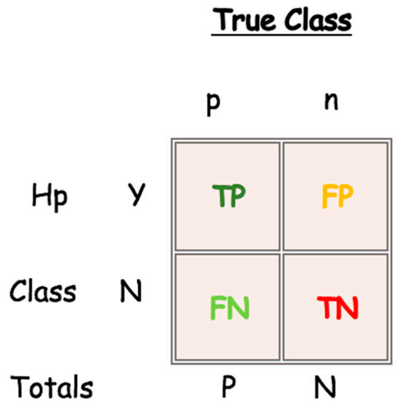

Figure S1. Confusion Matrix.

The Recall is defined as

$$Recall = \frac{Positives\ correctly\ classified}{Positivies} = \frac{TP}{P} = \frac{TP}{TP+FN} \tag{S10}$$

where positives and negatives mean class A and class B, respectively.

The Precision is defined as

$$Precision = \frac{Positives\ correctly\ classified}{Classified\ as\ positive} = \frac{TP}{Y} = \frac{TP}{TP+FP} \tag{S11}$$

The Accuracy is defined as

$$Accuracy = \frac{Correclty\ classified}{All} = \frac{TP+TN}{P+N} = \frac{TP+TN}{TP+FP+FN+TN} \tag{S12}$$

The negatives uncorreectly classified as positives is the False Positive Rate and is defined as

$$False\ Positive\ Rate = \frac{Negatives\ incorrectly\ classified}{Total\ negatives} = \frac{FP}{N} = \frac{FP}{FP+TN} \tag{S13}$$

The Informedness is defined as

$$Informedness = Recall - False\ Positive\ Rate \tag{S14}$$

References

1. Gentili, S.; Brondi, P.; Di Giovambattista, R. NESTOREv1.0: A MATLAB Package for Strong Forthcoming Earthquake Forecasting. *Seismological Research Letters* **2023**. https://doi.org/10.1785/0220220327.
2. Gentili, S.; Di Giovambattista, R. Forecasting strong subsequent earthquakes in California clusters by machine learning. *Physics of the Earth and Planetary Interiors* **2022**, *327*, 1-16, https://doi.org/10.1016/j.pepi.2022.106879.
3. Di Giovambattista, R.; Tyupkin, Yu.S. 2002. Burst of aftershocks as a manifestation of instability of the earth crust in an area of strong earthquake preparation. *In Proceedings of the European Seismological Commission (ESC*). XXVIII General Assembly. 1-6 September **2002**.
4. Gutenberg, B.; Richter, C.F. Earthquake magnitude, intensity, energy, and acceleration: (Second paper*). Bulletin of the Seismological Society of America* 1956, *46*, 105-145, https://doi.org/10.1785/BSSA0460020105.
5. Gentili, S.; Di Giovambattista, R. Pattern recognition approach to the subsequent event of damaging earthquakes in Italy, Physics of the Earth and Planetary Interiors 2017, 266, 1-17. https://doi.org/10.1016/j.pepi.2017.02.011 .
